# Morphological, Morphometric and Phaneroptic Variations of the Mediterranean Donkey and Tunisian Perspective on Conservation and Breeding

**DOI:** 10.3390/ani16020207

**Published:** 2026-01-09

**Authors:** Mohamed Aroua, Nour Elhouda Fehri, Antonella Fatica, Sana Khaldi, Samia Ben Said, Bayrem Jemmali, Mokhtar Mahouachi, Elisabetta Salimei

**Affiliations:** 1 Laboratoire d’ Appui à la Durabilité des Systèmes de Production Agricoles du Nord-Ouest, Ecole Supérieure d’Agriculture du Kef, Université de Jendouba, Complexe Universitaire Boulifa, Le Kef 7119, Tunisia; sabensaid@gmail.com (S.B.S.); taymallahmah@gmail.com (M.M.); 2 Department of Veterinary Medicine and Animal Sciences, University of Milan, Via dell’Università 6, 26900 Lodi, Italy; nour.fehri@unimi.it; 3 Dipartimento Agricoltura, Ambiente e Alimenti, Università degli Studi del Molise, Via de Sanctis snc, 86100 Campobasso, Italy; salimei@unimol.it; 4 Ecole Nationale de Médecine Vétérinaire Sidi Thabet, Université de Mannouba, Sidi Thabet 2020, Tunisia; sana.khaldi@gmail.com; 5 LR13AGR02, Mateur Higher School of Agriculture, University of Carthage, Mateur 7030, Tunisia; jemmali.bayrem@gmail.com

**Keywords:** donkey morphometrics, phaneroptic variation, sexual dimorphism, body measurements, local breeds

## Abstract

The domestic donkey (*Equus asinus*) remains an essential component of agricultural systems worldwide, particularly in rural Tunisia. This study aims to conduct a comprehensive morphological characterization of the Tunisian donkey population, focusing on phaneroptic variation, sexual dimorphism and live weight prediction. A total of 556 clinically healthy donkeys, consisting of 207 males and 349 females, were sampled from various governorates in Tunisia. Descriptive statistics revealed significant variability in withers height (98–147 cm), thoracic circumference (100–165 cm) and body length (86–144 cm). Principal Component Analysis identified coat color and belly pigmentation as major contributors to phaneroptic variation, explaining 69.556% of the total variance. Hierarchical Ascendant Classification further classified the population into three distinct groups, with Group I exhibiting smaller body dimensions, Group II having medium sizes, and Group III consisting of larger donkeys. Sexual dimorphism was detected in neck circumference (females: 64.30 cm *vs*. males: 61.97 cm; FDR-adjusted *p* = 0.0468). Regression models for predicting live weight indicated that thoracic circumference was the most reliable single predictor (R^2^ = 95.4%). Overall, the study documents a wide range of morpho-biometric variation within the Tunisian donkey population and provides practical tools for field-based weight estimation, offering valuable insights for future conservation strategies and management programs.

## Simple Summary

Donkeys are still essential to rural life in Tunisia, where they are used for farming, transport, and daily work. However, little information exists about their physical characteristics and differences between males and females. In this study, 556 healthy donkeys from different regions of Tunisia were measured to understand how their body shape and size vary. The results showed large differences in key body dimensions such as height, chest size, and body length. The analysis revealed three main groups of donkeys: small, medium, and large-sized animals. Female and male donkeys also showed some physical differences, particularly in neck and head measurements. The study developed a sidonemple equation to estimate the live weight of donkeys based on chest size, which can help farmers and veterinarians assess the animals’ condition without using a scale. Overall, these findings highlight the great diversity among Tunisian donkeys and provide useful information for improving their management and conservation.

## 1. Introduction

The domestic donkey (*Equus asinus*), despite its historical and socio-economic significance, remains an underrepresented species in the scientific literature. Since domestication, the donkey has played a transformative role in the development of transport systems across Africa and Asia, enabling the movement of goods and people and contributing to the growth of early urban settlements and pastoral societies [1,2]. However, the original reason behind its domestication remains a subject of scholarly debate. Current hypotheses suggest that initial domestication may not have been primarily driven by transportation needs but rather by other utilitarian purposes such as meat or milk production [3].

In Latin America the presence of donkeys dates to the post-Columbian era following the European colonization in 15th-century, during which these animals were introduced primarily as pack animals [4]. One notable breed, the Amiata donkey from Mount Amiata in Tuscany, Italy, is classified as endangered and represents a valuable case study for investigating the domestication history of donkeys. Morphologically, this breed exhibits similarities to ancestral subspecies such as *Equus africanus* and *Equus asinus somaliensis* [5]. Genetic studies, especially those based on mitochondrial DNA, support the hypothesis that modern donkeys descend from the African wild ass, with diverging lineages corresponding to *Equus africanus* and *Equus africanus somaliensis* [2,6,7]. However, the phylogenetic positioning of the North African domestic donkey remains unclear, although some researchers suggest a relationship with the Atlantic donkey population [8].

Globally, diverse donkey breeds reveal distinct domestication narratives. In Kenya’s Central Highlands, the native donkey population is genetically linked to the Nubian wild ass [9], while Bulgarian donkeys show ancestry from both Nubian and Somali wild asses [10,11]. In West Africa, the Kudu donkey predominates in northern Nigeria [12]. Serbia’s indigenous breeds include the Balkan and Banat donkeys [13], whereas the Czech Republic hosts a population of uncertain origin, but genetically related to *Equus asinus somaliensis*, *Equus africanus*, and other European breeds [14]. In Central Africa, donkey populations in Cameroon and Chad also exhibit Nubian ancestry [1,2]. In Latin America donkeys were introduced from the Iberian Peninsula by Spanish and Portuguese colonizers, and subsequently proliferated in regions such as Jamaica and the Peruvian coast, particularly in areas like Cañete [15].

The global donkey population is estimated at approximately 50 million animals, distributed across around 180 officially recognized breeds [16]. Geographically, their distribution is as follows: 1.6% in Europe, 42.6% in Asia, 38.7% in Africa, and 17% in the Americas. Only 12% of the global population resides in industrialized nations, while Africa (63%) and Asia (25%) hold the vast majority [16]. Notably, the growing demand for donkey hides in China primarily for the production of *ejiao*, a traditional medicinal gelatin has led to population declines in many countries [17]. In Tunisia the donkey population is estimated at 123,000 heads [18], but this figure likely underrepresents the actual numbers due to reporting gaps in rural regions [19]. As indigenous breeds face growing threats of extinction, the conservation of genetic resources has become increasingly urgent [20,21]. However, in contexts where genetic information remains scarce, fragmented, or unavailable, phenotypic characterization is widely recognized as a necessary and legitimate preliminary step. Comparative studies on morphological traits across populations are therefore essential for understanding phenotypic variation and for designing effective conservation and breeding strategies [22,23].

In this context, the present study aims to conduct a comprehensive morphological and morphometric characterization of Mediterranean donkeys, with a specific focus on the Tunisian population. More precisely, the study seeks to assess intra-population morphological diversity, qualitative traits, and derived morphometric indices to identify and describe the morphotypes present within the population. The developed models explaining the variation in both quantitative and qualitative animal traits could provide essential insights for breeding management and preservation of equine diversity in the Mediterranean region.

## 2. Materials and Methods

### 2.1. Study Area and Collaboration Framework

This research was conducted in close collaboration with the Society for the Protection of Animals Abroad (SPANA) and covered multiple governorates in Tunisia, including Le Kef, Jendouba, Kasserine, Siliana, Béja, Bizerte, Manouba, Kairouan, Nabeul, Monastir, and Zaghouan. These governorates encompass a wide range of agro-ecological zones from mountainous and semi-humid areas in the northwest (Le Kef, Jendouba, Béja), to semi-arid plains and steppe regions in the center (Kairouan, Kasserine), and coastal humid zones along the northeast (Nabeul, Bizerte, Monastir). Collectively, these regions account for over 80% of the national donkey population, according to the most recent data from ONAGRI [24]. These areas are predominantly characterized by marginal, traditional livestock farming systems, where donkeys continue to play an essential role in subsistence agriculture, notably in transporting goods and fetching water. This enduring reliance highlights the socio-economic and cultural importance of donkeys in rural Tunisian livelihoods and their integral function in the daily lives of agrarian communities. Although donkeys are widely distributed across Tunisia, there are no formally recognized regional breeds or region-specific local names for distinct donkey types. Instead, farmers often describe animals informally based on morphological characteristics.

### 2.2. Animal Selection and Sampling Criteria

A purposive sampling strategy was adopted, consisting of the deliberate selection of adult, clinically healthy working donkeys encountered during routine veterinary field activities conducted by the Society for the Protection of Animals Abroad (SPANA). This non-probabilistic approach was selected to ensure accurate and standardized morphometric assessments under field conditions and is widely employed in phenotypic characterization studies of working equids, where random population-wide sampling is logistically impractical.

A total of 556 donkeys (207 males and 349 females) were included in the study. Prior to enrolment, all animals underwent a standardized clinical examination performed by a qualified SPANA veterinarian. Only individuals with fully completed skeletal development were considered eligible. Donkeys were classified as adults at ≥5 years of age, based on dentition patterns and overall anatomical maturity, corresponding to the stage at which skeletal ossification is complete and morphometric proportions are considered stable in the species. The study population was defined according to explicit inclusion and exclusion criteria to ensure methodological consistency and reliable morphometric assessment. Inclusion criteria required that donkeys were adult animals aged at least 5 years, with completed skeletal and musculoskeletal development, clinically healthy, and actively used in agricultural work, transport, or traction. Eligible animals showed no signs of lameness, visible wounds, dermatological lesions, or systemic disease and presented a normal nutritional status at the time of examination. Exclusion criteria included animals younger than 5 years, due to ongoing skeletal growth, as well as animals older than 15 years, due to possible age-related alterations such as muscle atrophy, postural changes, and dental deterioration. Females in advanced stages of pregnancy were excluded to prevent pregnancy-related conformational changes from affecting morphometric measurements. Animals presenting clinical disorders or abnormal body condition were also excluded from the study.

Body condition was assessed using a 5-point donkey-specific Body Condition Score (BCS) scale, widely applied in working donkey populations. A normal body condition was defined as a BCS ranging from 2.5 to 3.5, whereas animals with BCS < 2 (emaciated) or BCS > 4 (over-conditioned) were excluded to minimize potential confounding effects of extreme nutritional status on morphometric traits.

All donkeys were handled using low-stress, humane procedures by experienced personnel. No invasive interventions were performed, and morphometric measurements were collected efficiently to minimize handling time and animal discomfort.

### 2.3. Morpho-Biometric Characterization

#### 2.3.1. Quantitative Variables

**Figure 1:**
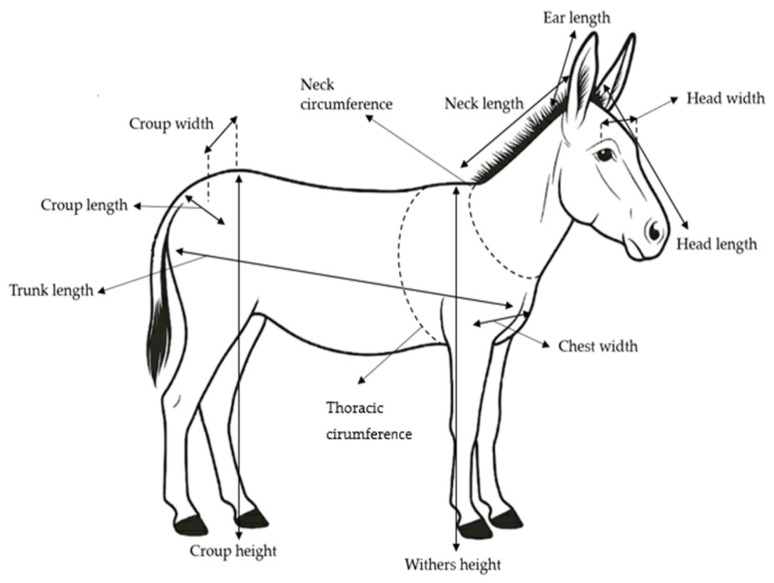
Schematic illustration of the morphometric measurements performed on the donkey’s body.

A standardized set of 15 body measurements was collected for each subject. Measurements strictly followed the protocols established by the FAO Animal Genetic Resources Characterization Guidelines [25], and were informed by international equid phenotyping studies [8,26,27]. All body measurements were taken by a single trained operator to reduce inter-observer variability (Figure 1). Each measurement was recorded three times on the same animal, and the mean of the three readings was used for analysis to ensure accuracy and repeatability. Data were obtained using calibrated instruments, including a digital livestock scale for live weight (PW 1500, BOSCHE GmbH & Co.KG, Damme, Belgium), a graduated flexible measuring tape for linear measurements, and a rigid double-beamed height gauge with millimetric precision for vertical dimensions. The recorded morphometric variables included live weight, withers height (vertical distance from the ground to the highest point of the dorsal interscapular region), thoracic circumference (circumference of the thorax measured immediately caudal to the scapulae, passing over the withers and behind the elbow joints), trunk length (distance from the point of the shoulder, at the scapulo-humeral joint, to the point of the buttock at the ischial tuberosity), neck length (distance from the cranial edge of the withers to the caudal margin of the mandible along the dorsal midline of the neck), left and right ear length (distance from the base of the ear at its insertion on the skull to the distal tip of the pinna), neck circumference (measured at the midpoint of the neck between the withers and the mandible), head width (distance between the most lateral points of the zygomatic arches), head length (distance from the occipital crest to the rostral extremity of the upper lip), chest width (horizontal distance between the lateral points of the scapulo-humeral joints), croup width (distance between the most lateral points of the tubera coxarum), croup length (distance from the sacral tuberosity to the ischial tuberosity), and croup height (vertical distance from the ground to the highest point of the sacral tuberosity).

These measurements were selected for their relevance in describing skeletal proportions, body mass, and overall conformation, which are critical for assessing functional capacity, adaptability, and productivity in working donkeys.

In addition to the primary body measurements, a set of derived morphometric indices was calculated to provide a more detailed assessment of body proportions, functional conformation, and adaptive traits in the Tunisian donkey population. These indices were grouped into three categories according to their biological significance: (i) productive indices, which evaluate thoracic development, body compactness, and overall robustness; (ii) functional indices, which assess biomechanical balance, locomotor efficiency, and structural proportionality essential for traction and work performance; (iii) ethological (adaptive) indices, which describe cranial and sensory characteristics linked to environmental adaptation and behavioral responsiveness.

All indices were computed as unitless ratios derived from the corresponding morphometric measurements, following formulas commonly used in equine and livestock morphometry formulas originally developed by Folch and Jordana [28] and later refined by Aroua [27] (Table 1). These ratios allow comparison among individuals regardless of absolute size differences and provide insight into the phenotypic structure of the population. The selected indices included measures of trunk–thorax relationships, thoracic and pelvic regions balance, cranial proportions, pelvic structure, and ear dimensions, offering a comprehensive framework for evaluating morphological diversity and functional suitability within the donkey population.

#### 2.3.2. Phaneroptic Characteristics

In addition to biometric measurements, five qualitative traits were observed in situ, following the criteria established in recent phenotypic characterization studies of North African donkeys [8,26]. These traits included coat color, muzzle pigmentation, belly color, limb coloration pattern, and the presence and pattern of dorsal and shoulder stripes.

These markers provide valuable data for breed identification, population differentiation, and ethnogenetic analyses. Furthermore, coat color and pattern traits often carry cultural, historical, or adaptive significance in traditional breeding systems.

### 2.4. Statistical Analysis

The collected data were entered into an Excel^®^ spreadsheet. Descriptive analysis, including coefficient of variation (CV), was performed to provide a detailed characterization of the body measurements of the sample. To examine both qualitative and quantitative traits, Principal Component Analysis (PCA) was conducted. Subsequently, a Hierarchical Ascendant Classification (HAC) was performed to group animals into distinct clusters and to generate a dendrogram illustrating similarity relationships among individuals. Because this study focuses on morphological and morphometric characterization, a Pearson correlation analysis was conducted to evaluate the degree of morphological harmony, defined as the strength and consistency of associations between different body measurements. Pearson correlation coefficients (r) were therefore calculated among all morphometric variables to assess proportional relationships within the population.

All body measurements were tested for statistical significance across groups using analysis of variance (ANOVA), with differences initially considered significant at *p* < 0.05. An independent-samples *t*-test was applied to assess sexual dimorphism by comparing mean body measurements between males and females. To account for the increased risk of Type I error associated with multiple comparisons across traits, all *p*-values derived from ANOVA and *t*-tests were adjusted using the Benjamini–Hochberg False Discovery Rate (FDR) correction. This adjustment allowed a more rigorous interpretation of statistical significance by reducing the likelihood of false-positive results.

To predict the weight of the animals, both linear and non-linear regression analyses were conducted using XLSTAT software 2016.02.27444 [30], enabling the establishment of predictive equations. This statistical approach allowed for the exploration of the relationships between various body measurements variables and the weight of the donkeys. Three linear regression models were developed to predict live weight based on body measurements, as shown below:

Model 1: Live weight=(thoracic circumference×4.20)+(withers height×3.02)−347.35
Model 2: Live weight=(thoracic circumference×4.22)−346.6
Model 3: Live weight=4.16×thoracic circumference+0.09×trunk length−350.88

More in detail, Model 1 incorporates both thoracic circumference and withers height as predictors. The inclusion of two variables enhances the overall explanatory power of the model, although it increases its complexity. Model 2, which includes only thoracic circumference as a predictor, its characterized by simplicity which makes it highly appealing for field applications, where ease of measurement and practicality are essential. Model 3 includes both thoracic circumference and trunk length as predictors.

## 3. Results and Discussion

### 3.1. Phaneroptic Characteristics

Among the studied donkey population, bay was the most prevalent coat color, accounting for 63.1% of all individuals (Table 2). This coat type is generally characterized by a pale or light base tone, often accompanied by gray shading around the muzzle and lips, and notably lacks primitive markings such as dorsal or shoulder stripes. These phenotypic traits are consistent with descriptions of the typical North African donkey phenotype as reported by [26,27,29].

The grey coat color was the second most frequent phenotype, observed in 22.5% of the sample (Table 2). This phenotype is characterized by the presence of both dorsal and ventral stripes, often forming a prominent cruciform pattern similar to the St. Andrew’s cross across the withers and shoulders. This morphological trait parallels that of the Nubian donkey, and also resembles phenotypes observed in donkey populations from northwestern Cameroon [31,32].

Donkeys with an all-black coat represented 14.4% of the population. These individuals generally exhibit a greyish ventral surface, sparse grey or white hair on the muzzle, and frequently display dorsal, ventral, or mixed stripe patterns. This coat type aligns with descriptions of specific European breeds, such as the Sicilian donkey and the Grand Berry donkey [27,33].

**Figure 2:**
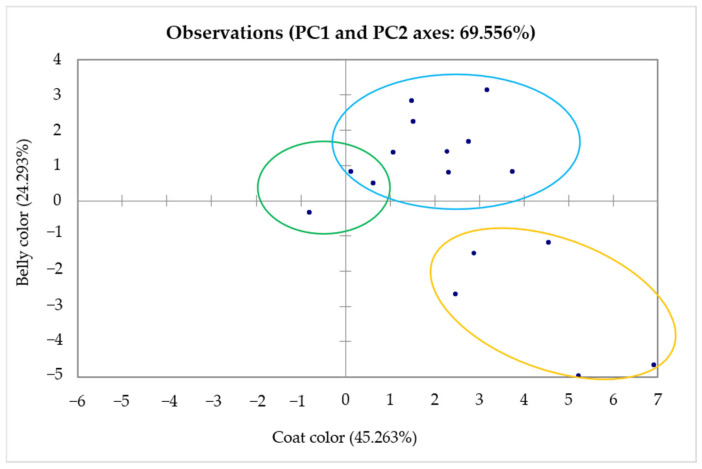
Principal component analysis of coat and belly colors in Tunisian donkeys (n = 556).

Principal Component Analysis performed on phaneroptic characteristics, with individuals and variables projected onto the first two principal components, accounted for 69.556% of the total variance, reflecting a strong discriminatory power of these two axes in capturing the main patterns of morphological diversity. The first principal component (PC1), which contributed 45.263% of the total variance, was primarily shaped by variation in coat color, indicating that this trait is the most influential factor in phaneroptic differentiation among the surveyed animals. The second component (PC2), representing 24.293% of the total variance, was largely associated with belly color, further enhancing the resolution of individual differentiation. The distribution of individuals across the PCA space was markedly heterogeneous, with wide dispersion along both axes, leading to the emergence of three distinct clusters (Figure 2). These groupings suggest the presence of well-defined morphotypes within the population, potentially reflecting underlying genetic substructures, historical selection pressures, or environmental adaptation mechanisms. The clarity of this structuring reinforces the relevance of coat and belly pigmentation as robust markers in donkey population studies [21,34]. Furthermore, the results highlight the usefulness of PCA as a powerful multivariate approach for phaneroptic classification, particularly in contexts where qualitative descriptors serve as proxies for deeper genetic and evolutionary processes [8]. These findings emphasize the importance of integrating morphological and pigmentation traits into broader frameworks for biodiversity assessment, breed conservation, and adaptive management strategies in equid populations.

The Hierarchical Ascendant Classification analysis reveals a clear hierarchical structure, as described below.

Group I, including 72.6% of the sampled individuals, represents the predominant cluster. Donkeys in this group are defined by a uniform bay coat, a gray-colored muzzle, absence of primitive markings (such as dorsal or ventral stripes), and a consistently gray belly. These features correspond closely to the characteristics of the traditional North African donkey, particularly the Tunisian breed [8,26,29,35]. The uniformity and prevalence of this group suggest it constitutes the dominant indigenous type widely distributed across the entire Tunisian territory, reflecting long-term adaptation to the diverse agroecological and agricultural conditions of the country.

Group II, representing 13.9% of the population, includes donkeys with dark coat pigmentation, predominantly all-black or gray markings, and a variable presence of primitive markings such as dorsal, ventral, or mixed stripe patterns. The distribution of these markings and the darker coloration aligns phenotypically with descriptions of the Grand Berry donkey or related European breeds [27,34]. The intermediate position of this group on the phylogenetic tree may reflect historical gene flow from imported or crossbred lineages, particularly in regions historically influenced by colonial or trans-Mediterranean exchanges. Group II is encountered more frequently in steppe and mountainous regions, particularly in the governorates of Kasserine, Kairouan, Kef, and Jendouba, where environmental pressures and local breeding practices may have favored this darker and more robust phaneroptic characteristics.

Group III, accounting for 13.5% of individuals, is characterized by a light gray coat and a distinctive dorsal and ventral striping pattern forming a St. Andrew’s cross, a primitive marking strongly associated with the Nubian donkey phenotype [1,31,36]. This group is found primarily in mountainous areas, where its distinctive stripe patterns and lighter coat may reflect functional or adaptive responses to the local environment. Although morphologically closer to Group II in several traits, Group III maintains clearly recognizable diagnostic markings that distinguish it as a separate cluster.

More in detail, Groups II and III exhibit a closer relatedness, which may reflect a shared ancestral lineage or convergent morphological adaptations. In contrast, Group I appear as phylogenetically more distinct, branching separately from the other two groups, which could result from an early divergence or a more isolated evolutionary trajectory within local agroecological contexts. This stratification is further supported by the profiles detailed in Table 3, outlining the diagnostic morphological traits characterizing each group.

Phaneroptic traits provide a valid and cost-effective approach for population differentiation in contexts where genomic data are limited. These insights provide a foundation for evidence-based conservation planning, emphasizing the importance of preserving not only genetic diversity but also the functional and phenotypic integrity of regionally adapted donkey types.

### 3.2. Body Measurements

The withers height of the studied Tunisian donkey population ranges from 98.0 cm to 147.0 cm, with an average of 115.2 cm (Table 4). This trait also presents a relatively low coefficient of variation (CV = 6.54%), indicating a high degree of uniformity in body stature across the population. This average height is notably higher than that observed in the Northwest Cameroon donkey (99.7 cm) [37] and in the Algerian donkey (114.0 cm) [8], both considered indigenous to the Sub-Saharan and North African regions. These results contradict earlier assertions by Wilson [38], who stated that donkeys’ withers height in Africa does not exceed 105.0 cm. The observed variation suggests that the body measurements traits of the Tunisian donkey population differ from those of donkeys native to Sub-Saharan Africa. This difference may be attributed to historical crossbreeding, selective breeding practices, and local adaptation processes, including influences from mule production and the introduction of external breeds during the colonial period [1,27,39]. In the broader regional context, Tunisian donkeys are taller than most African donkeys, including those from West Africa, Kenya, Ethiopia, and Morocco, but remain slightly shorter than the largest Mediterranean breeds, such as the Italian Pantesco [29,40,41,42,43]. This intermediate position reinforces the notion that Tunisian donkeys form a morphologically transitional group, bridging African and Mediterranean phenotypic patterns.

The average thoracic circumference of the Tunisian donkey (121.7 cm; Table 4) is comparable to that reported for donkeys in Cameroon (124.0 cm) [37], yet markedly exceeds the measurements documented for Senegal (108.9 cm) [44]. The CV for this trait (8.65%) confirms moderate uniformity in thoracic development, suggesting that thoracic circumference is a relatively stable characteristic within the population. This disparity reinforces the broader comparative pattern indicating that Tunisian donkeys consistently exhibit greater thoracic development and overall body length than most sub-Saharan African populations, whose morphometric profiles tend to reflect smaller and lighter animals. In contrast, the Tunisian measurements position this population closer to Mediterranean breeds, which are generally characterized by more robust body proportions [29,40,41,42,43]. Such enhanced thoracic dimensions in the Tunisian donkey likely result from long standing selective pressures, as local breeding practices historically favored individuals with greater strength and endurance traits essential for mule production and for performing demanding agricultural and transport tasks [19,45]. Beyond deliberate selection, these morphological traits may also reflect genetic drift and adaptive responses to Tunisia’s environmental context, where larger body size may provide advantages under local climatic and work-related conditions. Collectively, these factors contribute to the distinctive morphometric profile of the Tunisian donkey, which consistently occupies an intermediate position between African and Mediterranean phenotypic patterns.

In terms of neck morphology, the average neck length in the Tunisian donkey population (37.0 cm, Table 4) is shorter than that reported for the North Cameroon donkey (44.4 cm) [37] and the Ethiopian donkey (43.7 cm) [32]. This trait also shows a moderate CV (13.01%), indicating a higher degree of variability than major skeletal dimensions. The relatively reduced neck length in Tunisian donkeys could be a result of adaptation to the local topography, where shorter necks might be favored for maneuverability in agricultural tasks or for grazing in specific environments. Conversely, the neck circumference of Tunisian donkeys (63.6 cm, Table 4) aligns closely that of the North Cameroon donkey [37], suggesting similar muscular development in this region of the body, which is essential for physical strength and endurance in working donkeys.

The head width of the Tunisian donkey (23.1 cm, Table 4) is comparable to that of the Catalan donkey (23.0 cm) native to Spain [28]. The CV of this trait (14.36%) indicates moderate morphological dispersion, which may reflect cranial diversity influenced by historical gene flow. This similarity may reflect the historical introduction of the Catalan donkey to Tunisia in the 16th century, following the migration of the Moors to North Africa after the Reconquista [46]. The Catalan donkey, known for its robust and functional traits, was widely integrated into local breeding programs in Tunisia, contributing to the development of the modern Tunisian donkey phenotype. However, the head width of the Tunisian donkey is slightly smaller than that of the North Cameroon donkey (24.3 cm) [37], suggesting regional variations in cranial dimensions, which could be linked to genetic divergence or selective breeding for specific working traits.

As for the head length, the Tunisian donkey (44.7 cm, Table 4) is shorter than that of the Afar donkey (45.3 cm) and the Omo donkey (47.2 cm) from Ethiopia [32]. The remarkably high CV for this trait (22.20%) indicates strong phaneroptic variability, likely reflecting the coexistence of multiple morphotypes within the Tunisian population. This variability could also be associated with adaptive responses to arid and semi-arid environments, where a more compact head may facilitate thermoregulation and mobility in harsh climatic conditions.

The croup height of the Tunisian donkey population (118.9 cm, Table 4) exhibits a relatively low CV (8.78%), confirming uniformity in this major structural measurement. This value is higher than that of the Abyssinian donkey (95.4 cm) from Ethiopia [32], reflecting the greater body size and stature in the Tunisian donkeys. This difference in croup height further suggests that the Tunisian population exhibits larger overall body dimensions compared to other African breeds, potentially reflecting both selective breeding practices for size and genetic adaptation to specific work requirements that demand strong and larger physical structures.

The body index of 1.059 for the Tunisian donkey population, as reported in Table 5, indicates a well-developed body conformation that is suited for work-related tasks. This value aligns with those reported for donkeys from Northwest Cameroon [37] and the Catalan donkey breed from Spain [28], both recognized for their robust physical build and suitability for agricultural labor. The similarity in the body index values suggests that Tunisian donkeys share common morphometric characteristics with other highly functional donkey breeds from regions with similar ecological and work conditions.

In terms of the body profile index, the Tunisian donkeys exhibit an average value of 1.03 (Table 5), classifying them as brachymorphic with a convex profile. This classification is consistent with the North African and Southern European donkey breeds, as previously described by [27,47], which are known for their compact and strong body shapes that support physical endurance in strenuous tasks. Interestingly, both the Catalan donkey and the Northwest Cameroon donkey show a dolichomorphic profile, with a more elongated and narrow body shape, highlighting distinct regional adaptations. These differences may be linked to the variations in geographical environments, nutrition, workload, and heterogeneous management systems across regions. Furthermore, the hip to dock distance index of 0.76 (Table 5) supports the observation that the Tunisian donkeys generally possess a higher posterior than anterior, which is a key characteristic of functional donkeys used in manual labor and pack transport [48]. This conformation, often referred to as posterior dominant, facilitates better movement, stability, and strength when carrying heavy loads or navigating uneven terrain. The cephalic index, calculated at 0.55 (Table 5), places the Tunisian donkey population in the dolichocephalic category, meaning they have narrower, longer heads. This head shape is consistent with donkeys from North Africa and other regions where dolichomorphic traits are prevalent [27]. Algerian donkeys also display long heads (cephalic index ≈ 0.45), reinforcing this regional characteristic [29]. Additionally, the pelvic index of 0.90 (Table 5) suggests that the donkeys in this population exhibit a convex croup, which is a key feature in working donkeys, aiding in their ability to exert force efficiently during labor-intensive activities [49]. The combination of a dolichocephalic head and convex croup further supports the functional adaptation of these donkeys for agricultural and transport work in Tunisia [50].

The productive indices further support the robust conformation of the Tunisian donkey. The body index (1.06) and thoracic index (0.26) indicate a relatively deep and well-developed thoracic region, while the chest depth index (1.05) confirms a strong, capacious chest, a trait typically associated with good respiratory capacity and sustained work performance. These features are compatible with the historical use of donkeys in Tunisia for plowing, transport, and pack work in challenging rural environments. From a functional perspective, several indices highlight the structural balance and biomechanical suitability of these donkeys population. The relative body length and trunk length index (both with a mean of ≈0.98) show that trunk length is well proportioned relative to height, reflecting a balanced body frame rather than an excessively long or short trunk. The proportionality index (0.96) and the height-to-length ratio (1.02) further suggest that forequarters and hindquarters are in good equilibrium, avoiding extreme conformations that could compromise locomotion. The neck index (1.70) indicates a relatively thick and muscular neck compared with its length, a functional advantage for traction, load bearing, and control during work. The ear-length index (0.23) indicates relatively long ears in proportion to body height, which is typical of donkeys adapted to hot, arid climates, where enlarged ear surfaces facilitate thermoregulation and heightened sensory perception. Finally, the head-to-body index (0.39) reflects a moderate head proportion relative to trunk length, suggesting a good balance between sensory function and overall body mass.

Taken together, these morphometric indices highlight the functional suitability and adaptive value of the Tunisian donkeys. Their compact, brachymorphic profile, hindquarters-dominant conformation, convex croup, and dolichocephalic head morphology all converge toward a type that is well adapted to draft work, pack transport, and long-duration tasks in the Tunisian agro-ecosystems. These conformational features underscore the importance of preserving this locally adapted morphotype, as it embodies both the historical selection pressures and the ongoing functional needs of rural communities in Tunisia.

**Figure 3:**
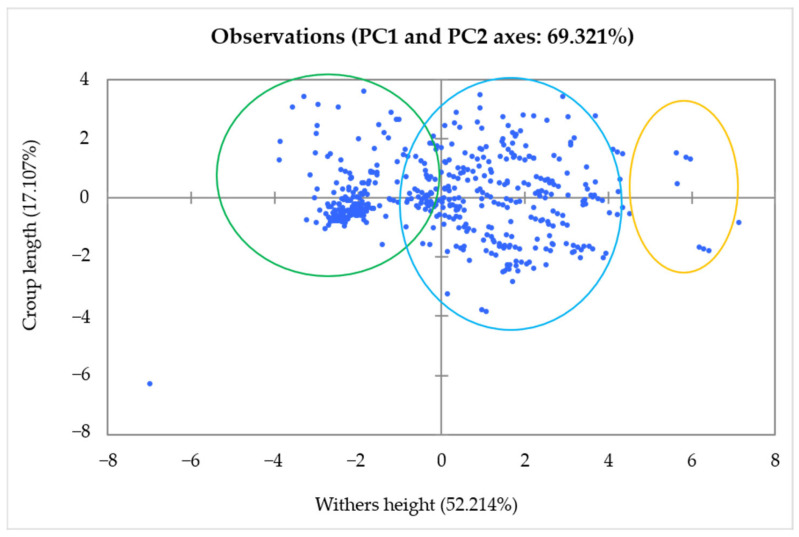
Principal component analysis of wither height and croup length in Tunisian donkeys (n = 556).

Principal component analysis of wither height (PC1) and croup length (PC2) demonstrates that the projection of observations onto the first two principal axes accounts for approximately 69.321% of the total variability, as illustrated in Figure 3. This variability is not uniformly distributed, with the first principal component explaining 52.214% of the variance, while the second principal component accounts for 17.107%. The distribution of the animals along these axes exhibits a notable spread, resulting in the formation of three distinct clusters or groups, highlighting the presence of significant variability in the population.

The body measurements characteristics of the three donkey groups are outlined in Table 6, reflecting significant differences in size and morphological characteristics among the groups. These differences are important for understanding the diversity within the donkey population, particularly in relation to environmental and genetic factors.

**Figure 4:**
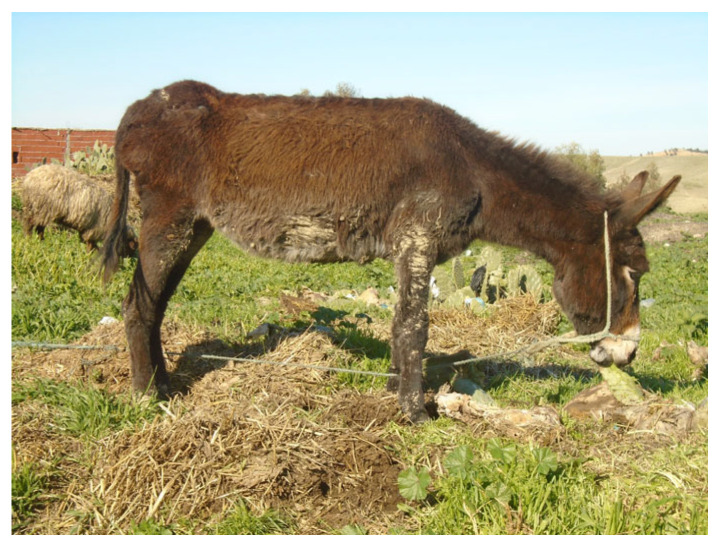
Representative donkey from Group I.

The first group (n = 293, Figure 4) consists of small-sized donkeys, characterized by an average withers height of 109.5 cm and a croup height of 112.6 cm (Table 6). These animals typically exhibit a bay coat, a common trait of the North African donkey [8,26,27,29]. A grizzled pattern is observed around the nostrils, lips, chin, and muzzle, in absence of stripes (Figure 4). Field observations indicate that this group is widely distributed across the entire Tunisian territory, reflecting its status as the dominant and most representative local morphotype.

**Figure 5:**
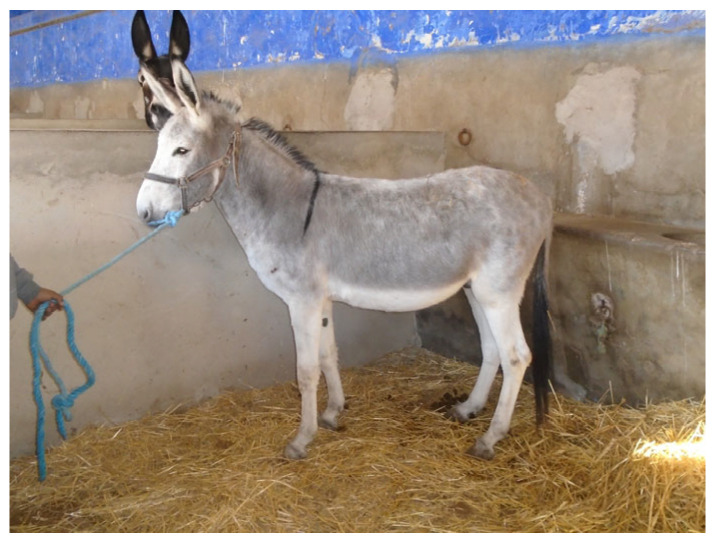
Representative donkey from Group II.

The second group (n = 171, Figure 5) includes medium-sized donkeys, with an average withers height of 120.8 cm and croup height of 125.7 cm (Table 6). These donkeys are distinguished by their gray coat, often displaying a characteristic dark band, a trait typical of the Nubian donkey [31]. Field observations indicate that this group is found predominantly in mountainous regions, particularly in the governorates of Kef, Jendouba, Zaghouan, and Béja, where local environmental conditions and management practices may have contributed to the persistence of this morphotype.

**Figure 6:**
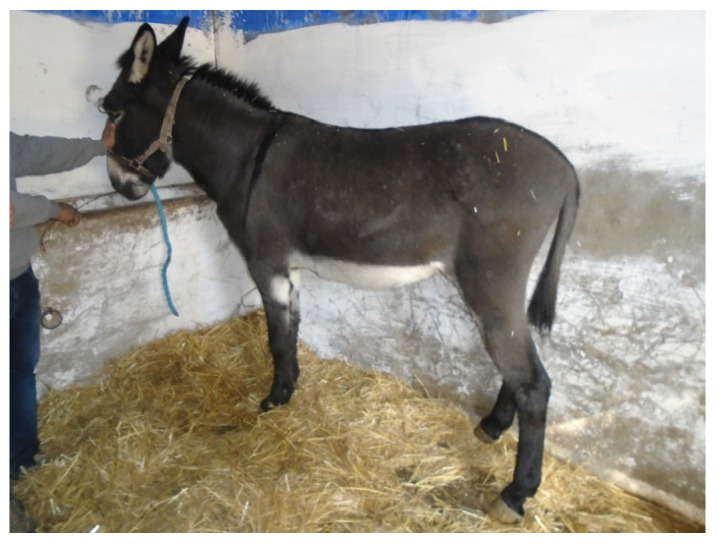
Representative donkey from Group III.

The third group (n = 52, Figure 6) comprises large-sized donkeys with an average withers height of 129.0 cm and croup height of 131.9 cm (Table 6). These donkeys exhibit a black coat and display dorsal, ventral, or mixed stripes commonly observed in Kasserine, where herds are selectively bred [19]. Field observations indicate that this group is primarily present in the steppe regions of Kasserine as well as in mountainous areas of Kef, Béja, and Jendouba, suggesting a distribution shaped by local environmental conditions and region-specific management systems.

Statistical analysis reveals that all variables differ significantly (*p* < 0.01) among the groups. Notably, Group III (large-sized donkeys) consistently shows the highest values for key body dimensions such as withers height, thoracic circumference, body length, and neck circumference (Table 6). These donkeys are considerably larger than both Group I (small-sized donkeys) and Group II (medium-sized donkeys), and the difference is significant and emphasizes the pronounced size variation. Additionally, Group III donkeys exhibit the largest head and neck dimensions, with an average head length of 43.9 cm and neck circumference of 67.4 cm, compared to Group I, which has an average head length of 39.0 cm and neck circumference of 58.7 cm. Group II falls between the two extremes, with intermediate values in most measurements (Table 6). The results also show significant differences (*p* < 0.001) in ear length, with Group III having the longest ears (30.0 cm on the left and 29.1 cm on the right), compared to Group I (24.9 cm on the left and 23.9 cm on the right) and Group II (28.9 cm on the left and 28.3 cm on the right). These morphological variations in ear size may be indicative of breed-specific traits and adaptations. Differences are also observed in croup dimensions (*p* < 0.001). Group III has the largest croup height (131.9 cm), croup width (30.3 cm), and croup length (38.5 cm), whereas Group I shows croup height of 112.6 cm, croup width of 32.9 cm, and croup length of 37.7 cm. Group II consistently exhibits intermediate values for all three croup measurements, further highlighting the morphological gradient among the groups.

### 3.3. The Variation in Body Measurements Between Male and Female Donkeys

The variation in body measurement between male and female donkeys of the local Tunisian population is presented in Table 7. Significant differences (*p* < 0.05) are observed only for neck circumference, with females showing a larger average value (64.30 cm) than males (61.97 cm) after applying the Benjamini–Hochberg correction for multiple comparisons (FDR-adjusted *p* = 0.0468). No other body measurements exhibited significant gender-based differences, as reported in literature [28,51,52], indicating that sexual dimorphism in this population is primarily expressed in neck circumference.

Moreover, the greater neck circumference in females could be linked to physiological and hormonal factors related to reproduction [52,53]. More in detail, it could reflect muscle development needed to support the demands of pregnancy, lactation, and other reproductive functions [53]. In many species, sexual dimorphism in neck musculature has been observed, with females often exhibiting enhanced musculature or other adaptations necessary for reproductive success [54]. Specifically, the withers height of males (115.07 cm) and females (115.05 cm) and the thoracic circumference measurements (males: 121.80 cm, females: 121.51 cm) are comparable, indicating similar body proportions across both genders (Table 7). This lack of significant variation in these traits may suggest that they are influenced more by environmental factors or breed characteristics, than by gender-specific biological differences. Although minor differences are observed in body length and croup height, they are not statistically significant (*p* > 0.05). The average body length in females (113.25 cm) is slightly greater than in males (110.59 cm), and similarly, the croup height in males (119.24 cm) is marginally higher than in females (118.14 cm) (Table 7). However, the lack of statistical significance in these measurements suggests that the overall body structure, in terms of length and croup height, remains relatively consistent between sexes. These slight differences could be attributed to individual variation or environmental influence, such as diet or growth conditions, rather than inherent sexual dimorphism [55].

The Pearson correlation analysis revealed clear and coherent relationships among the morphometric traits of the Tunisian donkey population, indicating strong structural harmony within the breed. Major body-size indicators such as withers height, thoracic circumference, and trunk length were strongly intercorrelated (r = 0.65–0.78, Table 8), demonstrating that donkeys with greater stature tend to exhibit proportionally larger axial and pelvic dimensions. Thoracic circumference showed the strongest association with live weight (r = 0.982), confirming its central role as the most reliable single predictor of body mass, in agreement with previous studies on equids [56,57]. Ear length traits (left and right ears) displayed a strong bilateral correlation (r = 0.976), but only moderate relationships with overall body size, suggesting that ear morphology varies partly independently from general body growth. Neck circumference showed moderate correlations with several size-related variables (r = 0.36–0.61), indicating that muscular and soft-tissue development follows, but is not strictly proportional to skeletal dimensions. In contrast, chest width, croup width, and secondary pelvic measures presented weaker correlations with the main body-size indicators (generally r < 0.30), highlighting greater independent variation in lateral body shape. These traits thus contribute uniquely to the phaneroptic variability within the population. The correlation structure suggests a generally harmonious morphometric profile with some traits exhibiting relative independence that may help differentiate morphotypes.

Taken together, these findings reinforce the importance of thoracic circumference as a practical and accurate predictor of live weight, as its strong correlation with body mass reflects the overall volume of the thoracic cavity and thus the general size of the animal [56,57]. Although withers height and trunk length were positively correlated with live weight, their weaker associations indicate that these traits, while informative of general body stature, do not capture mass variation as effectively as thoracic circumference. This pattern is consistent with the broader literature, which shows that linear skeletal measurements have lower predictive value for body mass compared with thoracic circumference in donkeys and other equids [14,58,59]. Overall, the correlation structure supports a harmonious morphometric profile, while highlighting specific traits that vary independently and may contribute to the differentiation among morphotypes.

**Figure 7:**
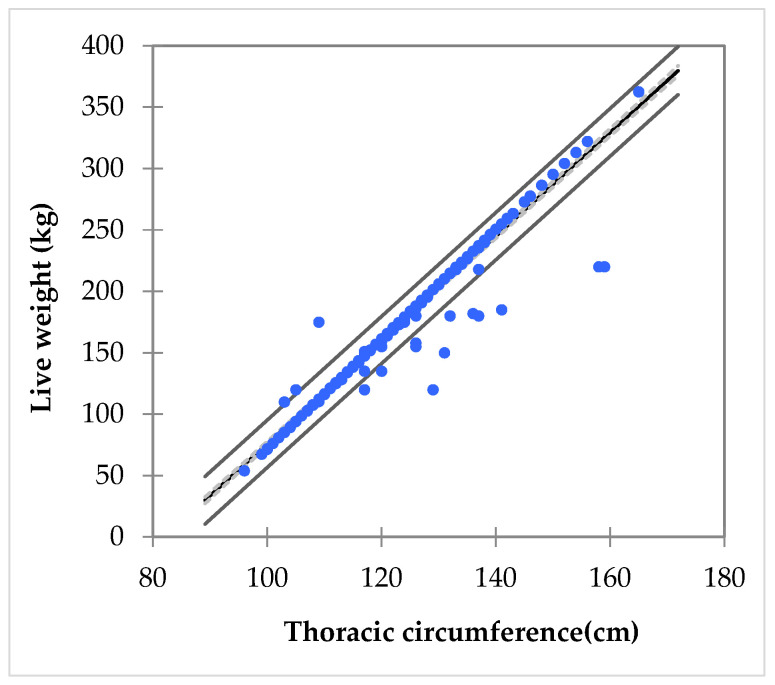
Linear regression of live weight on thoracic circumference in the Tunisian donkey population (n = 556). Blue dots represent individual observations, solid black line shows the predicted live weight from the regression model, dashed line indicates the 95% confidence interval of the mean prediction, while the outer solid lines represent the 95% confidence interval for individual observations.

Given the linear regression models developed to predict live weight from body measurements, all three investigated models demonstrate a high degree of fit to the data, with coefficients of determination (R^2^) of 0.954. Accordingly, Figure 7 shows the regression of live weight on thoracic circumference, along with the 95% confidence intervals for both the mean prediction and individual observations. This indicates that about 95.4% of the variability in live weight is explained by the models, confirming their robustness and predictive reliability.

**Figure 8:**
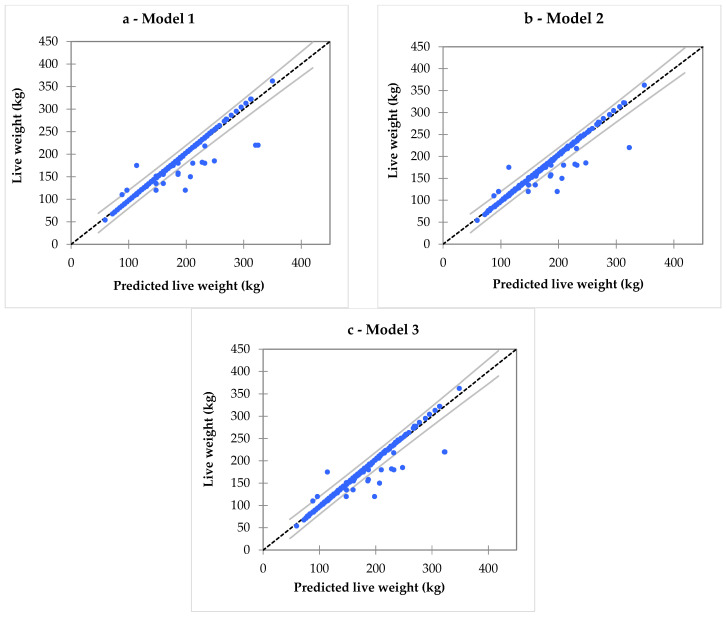
Linear regression of live weight on predicted live weight in the Tunisian donkey population (n = 556) based on Model 1 (**a**), Model 2 (**b**) and Model 3 (**c**). Blue dots represent individual observations, solid black line shows the predicted live weight from the regression model, dashed line indicates the 95% confidence interval of the mean prediction, while the outer solid lines represent the 95% confidence interval for individual observations.

Model 1, which includes both thoracic circumference and withers height as predictors, provides a high R^2^ (0.9543, Figure 8a), suggesting its usefulness when both morphometric variables are available. However, the added complexity may not be necessary in field conditions, where only one easily measurable morphometric variable is preferred. Model 2, including only thoracic circumference as a predictor, performs equally well, with the same R^2^ value (0.9537, Figure 8b). Its simplicity and high predictive accuracy make it highly effective and practical tool for estimating the live weight of Tunisian donkeys. Finally, Model 3, which includes thoracic circumference and trunk length, also achieves a comparable R^2^ value (0.9541, Figure 8c), but the inclusion of trunk length does not significantly improve predictive accuracy compared to Model 2. This indicates that thoracic circumference alone provides nearly all necessary information for estimating live weight. Consequently, in practical context where time, resources, and technical expertise are often limited, the simplicity and ease of application of Model 2 represent a clear advantage; moreover, it explains 95.4% of the variability in live weight.

## 4. Conclusions

This study provides a comprehensive morphometrical, morphological and phaneroptic characterization of the Tunisian donkey population, revealing substantial variation in pigmentation patterns and body dimensions. Multivariate analyses identified three coherent morphotypes differing in stature and phaneroptic traits, reflecting structured diversity without implying underlying genetic subdivisions. Morphometric variability was trait-dependent, with major skeletal measurements displaying notable uniformity, while several cranial, thoracic, and pelvic traits contributed disproportionately to population heterogeneity. Functional, productive, and ethological indices collectively described a compact and well-balanced conformation consistent with the species long-standing use in traction and load-bearing roles. Sexual dimorphism was limited, emerging significantly only in neck circumference. Correlation analyses demonstrated strong structural harmony among key body-size traits, confirming thoracic circumference as the most informative predictor of live weight.

Overall, these findings provide an evidence-based framework for phaneroptic monitoring and management of Tunisian donkeys, supporting conservation initiatives aimed at maintaining functional diversity and adaptive potential. Phaneroptic characteristics can offer practical value for future genetic improvement, but integration of molecular tools is required for the understanding of under-documented donkey population structure.

## Figures and Tables

**Table 1:** Morphometric indices: classification and calculation formulas.

Category	Index	Formula	Reference
Productive Indices	Body Index (BI)	Trunk Length/Thoracic circumference	[8,27]
	Thoracic Index (TI)	Chest Width/Thoracic circumference	[27]
	Chest Depth Index (CDI)	Thoracic circumference/Withers Height	[29]
	Body Profile Index (BPI)	Body Length/Withers Height	[27]
Functional Indices	Relative Body Length (RBL)	Trunk Length/Withers Height	[28]
	Proportionality Index (PI)	Withers Height/Croup Height	[8]
	Neck Index (NI)	Neck Circumference/Neck Length	[8]
	Pelvic Index (PeI)	Croup Width/Croup Length	[28]
	Height-to-Length Ratio (HLR)	Withers Height/Body Length	[28]
	Trunk Length Index (TLI)	Trunk Length/Withers Height	[8,29]
	Hip-to-Dock Distance Index	Hip-to-Dock Distance/Croup Length	[28]
Ethological (Adaptive) Indices	Cephalic Index (CI)	Head Width/Head Length	[27]
	Ear-Length Index (ELI)	Ear Length/Withers Height	[8]
	Head-to-Body Index (HBI)	Head Length/Trunk Length	[8]

**Table 2:** Frequency and percentages of observed qualitative traits in Tunisian donkeys (n = 556).

Variable	Categories	Number of Individuals	%
Coat color	Bay	351	63.1
	Gray	125	22.5
	Black	80	14.4
Muzzle color	Gray	522	93.9
	Black	34	6.10
Presence of stripes	No stripes	425	76.4
	Dorsal and ventral	81	14.6
	Ventral	28	5.00
	Dorsal	22	3.96
Belly color	Gray	511	92.0
	Uniform	45	8.00
Limb color	Light	534	96.0
	Spotted	22	4.00

**Table 3:** Phenotypic profile based on qualitative morphometric variables of the Tunisian donkey population (n = 556).

Group	Coat Color	Muzzle Color	Presence of Stripes	Belly Color	Limb Color
I	100% Bay	94% Gray, 6% Black	No stripes	100% Gray	81% Light, 19% Spotted
II	18% Gray, 82% Black	97% Gray, 3% Black	35% No stripes, 25% Ventral stripe, 30% Mixed stripes, 10% Dorsal stripe	80% Gray, 20% Uniform	100% Light
III	100% Gray	100% Gray	100% Dorsal and ventral stripes	72% Gray, 28% Uniform	64% Light, 36% Spotted

**Table 4:** Body measurements of the Tunisian donkey population (n = 556).

Variable (cm)	Minimum	Maximum	Mean	Standard Deviation	CV (%)
Withers height	98.0	147.0	115.2	8.568	6.54
Thoracic circumference	100.0	165.0	121.7	10.350	8.65
Trunk length	86.0	144.0	113.2	9.383	8.32
Neck length	28.0	66.0	37.0	4.804	13.01
Left ear length	24.0	38.0	26.7	3.137	10.96
Right ear length	23.0	37.0	25.9	3.231	12.56
Neck circumference	17.0	89.0	63.6	8.715	13.60
Head width	17.0	35.0	23.1	3.397	14.36
Head length	26.0	67.0	44.7	9.906	22.20
Chest width	24.0	45.0	26.5	5.359	20.25
Croup width	21.0	50.0	32.8	4.807	13.42
Croup length	20.0	69.0	39.1	7.823	20.68
Croup height	105.0	149.0	118.9	10.451	8.78

**Table 5:** Minimum, maximum, mean, and standard deviation (SD) of productive, functional, and ethological morphometric indices in the Tunisian donkey population.

Category	Index	Min	Max	Mean	SD
Productive Indices	Body Index (BI)	1.02	1.12	1.06	0.03
	Thoracic Index (TI)	0.18	0.45	0.26	0.18
	Chest Depth Index (CDI)	0.83	1.45	1.05	0.08
	Body Profile Index (BPI)	1.01	1.06	1.03	0.02
Functional Indices	Relative Body Length (RBL)	0.92	1.46	0.98	0.05
	Proportionality Index (PI)	0.65	1.40	0.96	0.09
	Neck Index (NI)	0.25	2.17	1.70	0.36
	Pelvic Index (PeI)	0.87	0.94	0.90	0.03
	Height-to-Length Ratio (HLR)	0.68	1.71	1.02	0.12
	Trunk Length Index (TLI)	0.58	1.44	0.98	0.13
	Hip to dock distance index	0.61	0.92	0.76	0.10
Ethological Indices	Cephalic Index (CI)	0.30	0.82	0.55	0.24
	Ear-Length Index (ELI)	0.16	0.39	0.23	0.02
	Head-to-Body Index (HBI)	0.18	0.70	0.39	0.06

**Table 6:** Body measurements (average values ± SD) among the three Tunisian donkey groups (n = 556).

Variables (cm)	Group I (n = 293)	Group II (n = 171)	Group III (n = 52)	*p*-Value	FDR-Adjusted *p*-Value
Withers height	109.5 ± 4.80	120.8 ± 4.66	129.0 ± 6.87	0.0010	0.001
Thoracic circumference	117.1 ± 7.44	124.2 ± 8.40	140.1 ± 6.63	0.0012	0.002
Body length	107.8 ± 5.90	117.1 ± 5.62	131.3 ± 5.48	0.0016	0.002
Neck length	36.0 ± 4.04	38.5 ± 5.78	38.3 ± 3.59	0.0045	0.008
Left ear length	24.9 ± 1.99	28.9 ± 2.73	30.0 ± 2.33	0.0003	0.001
Right ear length	23.9 ± 1.99	28.3 ± 2.73	29.1 ± 2.36	0.0004	0.001
Neck circumference	58.7 ± 6.01	71.0 ± 7.46	67.4 ± 6.22	0.0002	0.001
Head width	22.3 ± 2.74	23.9 ± 4.07	25.0 ± 2.92	0.002	0.004
Head length	39.0 ± 7.31	54.6 ± 5.12	43.9 ± 8.82	0.0007	0.002
Chest width	24.6 ± 3.52	28.8 ± 5.87	29.6 ± 7.43	0.006	0.011
Croup width	32.9 ± 4.01	33.3 ± 5.20	30.3 ± 6.64	0.001	0.003
Croup length	37.7 ± 6.15	41.7 ± 9.90	38.5 ± 6.45	0.003	0.006
Croup height	112.6 ± 8.32	125.7 ± 5.71	131.9 ± 6.96	0.0001	0.0005

FDR-adjusted *p*-value: *p*-values adjusted using the Benjamini–Hochberg False Discovery Rate (FDR) correction.

**Table 7:** Sexual dimorphism in body measurements (average values ± SD) of the Tunisian donkey population (n = 556).

Variables (cm)	Males	Females	*p*-Value	FDR-Adjusted *p*-Value
Withers height	115.07 ± 3.5	115.05 ± 2.9	0.972	0.972
Thoracic circumference	121.80 ± 4.3	121.51 ± 4.2	0.481	0.5686
Trunk length	110.59 ± 3.8	113.25 ± 4.2	0.064	0.2773
Neck length	36.68 ± 1.2	37.10 ± 1.5	0.088	0.1907
Left ear length	26.90 ± 1.4	26.70 ± 1.0	0.312	0.4056
Right ear length	25.72 ± 1.5	25.90 ± 1.2	0.284	0.4615
Neck circumference	61.97 ± 1.2	64.30 ± 1.5	0.0036	0.0468
Head width	23.90 ± 0.5	22.86 ± 0.66	0.0080	0.0520
Head length	43.50 ± 1.8	45.20 ± 1.9	0.071	0.2308
Chest width	25.96 ± 1.7	26.70 ± 1.3	0.112	0.2080
Croup width	33.10 ± 1.2	32.61 ± 1.6	0.164	0.1773
Croup length	39.21 ± 0.9	39.06 ± 1.1	0.298	0.4302
Croup height	119.24 ± 2.2	118.14 ± 1.9	0.076	0.1976

FDR-adjusted *p*-value: *p*-values adjusted using the Benjamini–Hochberg False Discovery Rate (FDR) correction.

**Table 8:** Pearson correlation coefficients of donkeys body measurement (r values).

Variables	WH	TC	TL	NL	LEL	REL	NC	HW	HL	CW	CrW	CrL	CrH	LW
Withers height (WH)	1													
Thoracic circumference (TC)	0.654	1												
Trunk length (TL)	0.778	0.658	1											
Neck length (NL)	0.343	0.298	0.303	1										
Left ear length (LEL)	0.651	0.496	0.561	0.401	1									
Right ear length (REL)	0.644	0.503	0.558	0.420	0.976	1								
Neck circumference (NC)	0.576	0.297	0.362	0.229	0.595	0.611	1							
Head width (HW)	0.163	0.025	0.271	0.182	0.242	0.247	0.321	1						
Head length (HL)	0.395	0.122	0.181	0.131	0.588	0.608	0.593	0.087	1					
Chest width (CW)	0.016	−0.044	−0.085	0.116	0.083	0.066	0.174	0.225	0.185	1				
Croup width (CrW)	0.268	0.025	0.218	0.215	0.289	0.308	0.365	0.572	0.176	0.135	1			
Croup length (CrL)	0.143	−0.020	0.059	0.156	0.179	0.193	0.376	0.384	0.149	0.469	0.389	1		
Croup height (CrH)	0.804	0.556	0.666	0.343	0.599	0.598	0.493	0.132	0.423	0.177	0.130	0.163	1	
Live weight (LW)	0.644	0.982	0.652	0.294	0.476	0.484	0.281	0.035	0.095	−0.045	0.036	−0.013	0.543	1

## Data Availability

The raw data supporting the conclusions of this article will be made available by the authors on request.
